# Organ of Calculation

**Published:** 1845-01

**Authors:** 


*Organ of Calculation.*—Vermont has furnished two or three boys, within the last twenty-five years, whose sagacity for arithmetical pursuits was of an extraordinary character. The autobiography of the far-famed Zera Colburn is familiar to the public. After having positively astonished the mathematicians, both here and in Europe, with the rapidity, accuracy and mystery with which he conducted the most elaborate arithmatical calculations, all at once, equally to the surprise of himself and every body else, he actually lost the faculty of doing wonders in figures. No effort on his part was successful in recovering a power that made his name ring over the world as an eighth wonder.

Another calculating boy, by the name of Safford, now only eight years of age, says the Vermont Journal, has been discovered in Vermont, who will give the product of four figures by four, performing the operation mentally nearly as quick as one can do it with pen and paper. He has also multipled five places of figures by five, which was the extent of Zera Colburn's powers in his best days. He will extract the square and cube roots of numbers extending to nine or ten places, performing the operation quite rapidly in his head. The division of numbers into their factors is a favorite amusement with him. Give him the age of a person, and he will give the number of seconds correctly.

How can the doctrines of the phrenologist be called in question, with such sustaining proofs of their truth, as are presented in this and many other analogous cases?

*Boston Med. and Surg. Jour.*

